# Milestones-based direct observation tools in internal medicine resident continuity clinic

**DOI:** 10.1186/s12909-017-1077-y

**Published:** 2017-12-04

**Authors:** Jonathan P. B. Berz, Teresa Cheng, Lisa M. Quintiliani

**Affiliations:** Boston University School of Medicine/Boston Medical Center, 801 Massachusetts Avenue, Crosstown 2, Boston, MA 02118 USA

## Abstract

**Background:**

With milestones-based assessment, there is an increased need for tools to facilitate direct observation of clinical trainees. This study was designed to compare a Mini-CEX tool to new direct observation tools (DOTs) linked to internal medicine milestones.

**Methods:**

A web based survey was used to examine satisfaction and usefulness of DOTs compared to the Mini-CEX. Residents and preceptors were surveyed three times over 6 months with half serving as control (using mini-CEX) compared to those using the DOTs. Likert scale quantitative answers and qualitative comments were analyzed using generalized estimating equations.

**Results:**

Out of 94 residents and 32 faculty 81 and 90% completed the survey for at least one time point. In adjusted models, there was no significant change in resident evaluation comparing the tools on a number of questions including overall satisfaction and resident perception of receiving high quality feedback. By contrast, faculty preceptors reported increased ratings on many of the questions evaluating their use of the new tools including ability to provide higher quality feedback and overall satisfaction.

A number of challenges and benefits of the new tools were identified in qualitative feedback by both residents and preceptors.

**Conclusions:**

All parties recognized the value and limitations of direct observation. Overall these new office based DOTs were perceived similarly to the mini-CEX by residents while faculty reported higher satisfaction. The DOTs are a useful addition to the tool box available for the assessment of clinical skills of medical trainees, especially from the viewpoint of faculty preceptors.

**Electronic supplementary material:**

The online version of this article (10.1186/s12909-017-1077-y) contains supplementary material, which is available to authorized users.

## Background

Getting into the exam room to directly observe resident clinical performance has long been recognized as an important method of assessing internal medicine resident competence. Faculty gain important insight into trainee clinical competence and communication skills that could not otherwise be obtained. With the introduction of milestones-based evaluation, internal medicine residency programs have an even greater need for direct observation of resident clinical care to inform the evaluation process [1]. The American Board of Internal Medicine mini-clinical evaluation exercise (Mini-CEX) has been well established as an effective tool to facilitate brief observation of trainee clinical care coupled with feedback after the encounter [2]. However, because Mini-CEX tools can be general in nature and may in fact measure clinical competence overall rather than any areas of competency specifically, programs are working to create tools that are more closely linked to the internal medicine reporting milestones [3]. Indeed in our program, the mini-CEX tools used were general in nature and did not specifically address milestones as the newly designed DOTs do.

Especially in large programs where clinical competency committee members will not likely have had the opportunity to interact with all resident trainees, clinical educator faculty need tools to assess milestone competency based on interactions with residents. Others have described the importance of direct observation of the internal medicine curricular milestones as a way to assess residents’ competence in performing Entrustable Professional Activities (EPAs) [4].

In this study, we aimed to evaluate faculty preceptors’ and internal medicine residents’ perceptions of satisfaction and usefulness of a pre-existing set of general Mini-CEX tools compared to four newly created milestone-based direct observation tools (DOTs) in a primary care continuity clinic setting. Findings from this study can be used to inform the selection and implementation of evaluation tools for internal medicine residents in the future.

## Methods

### Study population

Participants were residents in the Boston Medical Center Internal Medicine Residency program and associated primary care faculty who had continuity clinic at the program’s main hospital based clinic site. Boston Medical Center is a large, urban safety net hospital. Resident clinic was held in the hospital ambulatory building on two adjacent floors, which were similar in terms of patient characteristics, who are typical for a large inner city based primary care academic practice. Residents are evenly distributed between floors by post graduate year and are placed based on program scheduling needs rather than preference or any particular career orientation (such as primary care). On average there are 6 residents and 2 preceptor faculty per precepting session. However there is variation and some clinical sessions have as many as 9 residents and 3 preceptors working together.

### Study design

In December 2013, the DOTs were introduced on one floor of the hospital ambulatory building, while residents and faculty on a second floor continued to use the Mini-CEX tools(the control). Data collection took place at time 1-baseline (December 2013), time 2 (March 2014), and time 3 (June 2014). At time 2, (March 2014), the DOTs were introduced on the second floor so that all residents and faculty on both floors were using primarily the DOTs from then on. At this point while the mini-cex tools were not prohibited from being used they were no longer readily available for use and there was a strong push to use the DOTs. The Boston Medical Center Institutional Review Board determined the study to be exempt.

### DOT intervention

The DOTs used in this study were adapted from tools originally created at the University of New Mexico Internal Medicine Residency Program. Each of the four tools created are based on commonly occurring situations in the primary care resident continuity practice of our hospital. Four versions were created based on the following common ambulatory situations: 1) Explaining a test result; 2) Initiating a controlled substance contract; 3) Recommending an age appropriate screening test; and 4) Counseling for lifestyle habits (diet, alcohol, exercise, etc.,). (see Additional file 1).

Faculty were directed on how to use the new tools in 1 h faculty development sessions and in email communication. Residents were alerted to the new tools through program wide informational emails and by their preceptor faculty. During the ambulatory block week – one of every 4 weeks - residents were instructed to ask preceptors to complete DOTs and give feedback based on the observation. Periodic emails were sent to all residents and faculty reminding them to complete at least one DOT per week of ambulatory clinic block.

### Measurements

Separate faculty and resident web-based surveys were developed using the site www.hostedsurvey.com. Each study participant was linked to the survey using a number in order to track the number of surveys completed by each participant. Only the study PI had access to the names associated with each number though this was not accessed at any time during the study. Faculty and residents were asked to respond to the questions thinking about the forms (the mini-CEX or the DOTs) they were using at the time of the survey. Participants who completed all surveys were entered into a raffle for two gift cards.

Residents responded to a 10-item survey, which assessed gender; year of training; future career plans (outpatient or inpatient general internal medicine versus subspecialty or other); future practice setting (academic, private/group practice, or other); perceived quality of preceptor feedback (“I receive high quality feedback from my preceptors using the mini-cex observation tool” answered on a 5-point scale from 1: Strongly disagree to 5: Strongly agree); the mini-CEX tool’s usefulness in receiving feedback on 4 communication-related attributes (general communication, empathy towards patients, use of patient centered strategies, and skill in conveying risks and benefits to patients, all answered on a 5-point scale from 1: Strongly disagree to 5: Strongly agree); and overall satisfaction with the mini-CEX tool (5-point scale from 1: Strongly disagree to 5: Strongly agree). In addition the survey contained an area where residents could give free text comments regarding limitations and advantages of the currently available methods of receiving feedback on their performance in clinic. These open-ended questions served to supplement the responses with more in-depth qualitative data. These same survey questions were used in assessing those using the new DOTs as well.

Faculty responded to a 13-item survey which was pilot tested with a small group of three core residency faculty. Questions included gender; years of being a preceptor; perceptions of ease of use of the mini-CEX (5-point scale from 1: Strongly disagree to 5: Strongly agree); perceptions of ability to give high quality feedback with the mini-CEX (5-point scale from 1: Strongly disagree to 5: Strongly agree); usefulness of the tool in evaluating resident communication skills, demonstration of empathy, use of patient centered strategies and skills in conveying risks/benefits of test and treatments, all answered on a 5-point scale from 1: Strongly disagree to 5: Strongly agree; and overall satisfaction with the tool (5-point scale from 1: Strongly disagree to 5: Strongly agree). At the end of the survey, preceptors had the opportunity to write comments about limitations and advantages of the currently available methods of evaluating residents in clinic. Again this question served to supplement the quantitative data.

### Analysis

Generalized estimating equations (GEE) were used to assess differences in responses between the intervention and control across the three time points. Response scores on the 5 point Likert scale were analyzed as continuous variables. Intervention with DOT was compared with control. The GEE analysis method made it possible to include all participants who completed at least one survey time point because the method counts each observation separately. The beta estimates reported are effect sizes which represent differences between groups on the Likert scale. Both unadjusted models and models adjusted for post graduate year, gender, medical specialty, practice setting (for residents), gender and number of years as a preceptor (for preceptors) were conducted. SAS statistical computing software, version 9.3, was used.

Open-ended/free text responses were analyzed using a general inductive data analysis approach. This began by first systematically reading the responses. One investigator generated a series of codes to represent themes found in the text; these codes were then applied to the text and discussed among two investigators. We noted any areas of uncertainty or where the code was misapplied and developed consensus on the coding framework. The final codes were then applied to all of the free-text responses. Codes were then summarized into themes and presented in the results section.

## Results

### Resident survey

**Fig. 1:**
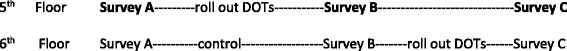
Study timing diagram

Out of 94 residents who have continuity clinic at the main hospital site and were eligible to participate in the survey study, a total of 76 (81%) completed the surveys for at least one of the time points. Specifically the number of residents who completed a survey at each time point by floor is as follows: 5th floor (Time point A, B, C: *n* = 23, *n* = 29, *n* = 19); 6th floor (Time point A, B, C: *n* = 31, *n* = 33, n = 33). (Figure 1 & Table 1) During the 4-month study time period a total 78 of the new DOTs were used- 36 of these were for lifestyle counseling, 18 for explaining a test result, 17 for discussing a screening test and 7 for having a discussion related to a controlled substance contract. Overall, there were few differences between unadjusted and adjusted models for residents and preceptors, indicating no confounding by the included variables. Findings from adjusted models are reported below.

**Table 1:** Number of respondents by time point & floor

Floor	Survey Time Point		
	A	B	C
Residents			
5th floor	23	29	19
6th floor	31	33	33
Preceptors			
5th floor	10	10	10
6th floor	9	10	8

*As depicted in the study time line (Fig. 1) 5th floor respondents were using only mini-CEX at time point A and DOTs at time points B &C and 6th floor survey respondents were using mini-CEX tools at time points A & B and DOTs at time point C

Residents who completed the baseline survey were 43% female, were spread across PGY1, 2 and 3 years (27.8%, 29.6%, 42.6). The majority of residents (78%) identified themselves as planning to enter a subspecialty after residency, while 20% predicted they would have a generalist career. Overall, there was no difference in change of resident perception of receiving high quality feedback comparing the general Mini-CEX tools with the new milestones-based DOTs. Similarly there was no difference in usefulness of feedback on 3 of the traits which the tools were designed to assess - communication skills, demonstration of empathy towards patients and use of patient centered strategies. Overall satisfaction with use of the tools was unchanged comparing the new DOTs with to the old Mini-CEX. There was a statistically significant increase in perceived usefulness in receiving feedback on skills conveying risk and benefits of tests and treatments when using the DOTs (a difference of 0.31 on a 5 point Likert scale), though this effect was no longer significant in the adjusted model (Table 2).

**Table 2:** Effect of DOT intervention compared to mini-CEX as measured by 5 point Likert scale- Residents

	Unadjusted *n* = 76*		Adjusted *n* = 75**	
Question	Crude β (95% CI)	*p*-value	Adj. β (95% CI)	*p*-value
I receive high quality feedback from my preceptors.	0.06 (−0.20, 0.31)	0.66	0.05 (−0.21, 0.31)	0.68
Overall, I am satisfied with the direct observation tools currently available in clinic	−0.12 (−0.39, 0.15)	0.38	−0.13 (−0.40, 0.15)	0.36
The tools are useful in receiving feedback on: Communication skills in general	0.09 (−0.20, 0.38)	0.54	0.14 (−0.16, 0.43)	0.35
Useful in receiving feedback on: Demonstration of empathy towards patients	0.13 (−0.18, 0.44)	0.41	0.15 (−0.17, 0.47)	0.36
Useful in receiving feedback on: Use of patient centered strategies	0.21 (−0.06, 0.48)	0.13	0.22 (−0.05, 0.50)	0.11
Useful in receiving feedback on: Skill conveying risks & benefits of tests & treatments	0.31 (0.01, 0.61)	0.04	0.29 (−0.00, 0.59)	0.05

Model adjusted for post graduate year, gender, subspecialty versus general medicine career orientation and academic setting versus private practice career plans

We examined residents’ responses (*n* = 69 responses) to the open-ended questions on the resident survey. At time 1, when residents responded to the survey had been using the Mini-CEX tools, time, observations, preceptor availability and artificiality were the predominant themes expressed by the residents. Overall, low value was ascribed to the observations. For the resident survey completed after the introduction of the new DOTs, similar themes of time, flow, observation and preceptor issues were reported. Residents specifically mentioned the DOT tool in their surveys and a majority perceived a low value to the observation forms. However the frequency of observations in particular seemed to be negatively viewed, which is understandable in the setting of time, flow and preceptor availability. Residents also acknowledge the narrow scope of the tools limited their ability to find a suitable patient for observations. Residents however, did express positive statements, perceiving the DOTs to be more realistic, helpful for communications in ambulatory medicine, and provided valuable feedback (see Table 4 with representative quotes).

### Preceptor survey

Out of 32 preceptor faculty associated with the residency program who were eligible to participate, 28 (90%) preceptors completed at least one survey. Specifically the number of preceptors who completed a survey at each time point by floor is as follows: 5th floor (Time point A, B, C: *n* = 10, n = 10, n = 10); 6th floor (Time point A, B, C: *n* = 9, n = 10, n = 9) (see Fig. 1 & Table 1). Preceptors perceived the new tools as somewhat easier to use compared to the old tools (beta = .64, *p* = 0.003) and reported that they were able to provide higher quality feedback using the new tools compared to the old tools (beta = 0.60; *p* = .006). The new tools were more useful in evaluating residents on communication skills (beta = 0.43, *p* = .002),and patient centered strategies (beta = 0.67, p = .002). However there was no difference between tools in evaluating empathy or conveying risks and benefits of tests and treatments or in perception of providing adequate information to assess residents. Additionally preceptors reported they were more satisfied with the new tools compared to the old tools (beta = 0.78, *p* = .001). The beta estimates reported represent the average difference in the 5-point Likert scale between the tools being compared (Table 3).

**Table 3:** Effect of DOT intervention compared to mini-CEX as measured by 5 point Likert scale- Preceptors

	Unadjusted *n* = 28*		Adjusted n = 28**	
Question	Crude β (95% CI)	*p*-value	Adj. β (95% CI)	*p*-value
The tools available are easy to use	0.60 (0.21, 0.99)	0.004	0.64 (0.25, 1.04)	0.003
I am able to provide high quality feedback to residents	0.58 (0.21, 0.94)	0.003	0.60 (0.19, 1.01)	0.006
Usefulness in evaluating communication skills in residents	0.44 (0.18, 0.69)	0.001	0.43 (0.17, 0.69)	0.002
Useful in evaluating demonstration of empathy toward patients	0.06 (−0.23, 0.35)	0.68	−0.03 (−0.30, 0.25)	0.85
Useful in evaluating resident use of patient centered strategies	0.65 (0.25, 1.05)	0.002	0.67 (0.26, 1.08)	0.002
Useful in evaluating resident on conveying risks and benefits of tests and treatments	0.17 (−0.12, 0.46)	0.24	0.07 (−0.20, 0.34)	0.60
Overall, I am satisfied with the tools	0.74 (0.34, 1.14)	0.001	0.78 (0.33, 1.22)	0.001

Adjusted for gender and # of years working as a resident preceptor

We examined preceptors’ responses (*n* = 33 responses) to the open-ended questions on the preceptor survey. Before introduction of the new DOTs, the preceptors acknowledged the challenges of time, preceptor availability and flow to observation in ambulatory clinic. They also commented on the artificial nature of the Mini-CEX observations but were more concerned about the contrived and scripted aspects of resident performance. Regarding the older CEX tool, preceptors often perceived it to be of low value and noted deficiencies in it being too general. Many commented that the CEX tool worked more as a general way to get into the examination room to provide direct observations than its value as a particular tool. After implementation of the new DOTs, additional feedback from the preceptors were that the scope of the tools were too narrow and it was challenging to find the right clinical situations for its use. However, there were no negative comments on the specific tools and many preceptors were pleased with the “progress on observation and feedback tools” (see Table 4).

**Table 4:** Representative responses to open ended survey question soliciting general comments

Category	N^a^	Comment
Sample resident quotations *before* introduction of new tools		
Observation	13	Rarely do my preceptors directly observe me, and if observed it is only in the final discussion in clinic after I have done the initial evaluation. I often don’t know in advance if a patient will have questions or an emotional response I need to respond to, so I can’t tell my preceptor when to observe me
Value	5	Although we are all short on time and therefore having a more involved evaluation system would probably be a burden for all, I believe that the current feedback system is moderately insufficient. Typically, a preceptor will agree to fill out a mini-cex, observe 1–2 min of a patient interaction and either hand the form back to you or to the program office without reviewing it with you. Most of the time, these forms are just check boxes or 1-5 scales and have little substantive feedback. It would be nice to have a more involved feedback system as I feel that feedback is a significant part of the training process.
Preceptor availability	9	Can be very difficult to work the mini-CEX into a busy clinic because it takes away one of the preceptors from the rest of the group. It ends up causing other people to wait and backing up the clinic*.*
Artificial	8	I think the mini-cex is too limited an evaluation to properly assess how we interact with patients. There is so much to the interaction that is not observed. Additionally, when someone is observing, it interferes with rapport and trust, as the patient is not really sure why we’re being supervised and may doubt our abilities.
Sample resident quotations *after* introduction of new tools		
Value	10	The DOTs provide useful feedback. However, clinic is often very busy and I feel that the expectation to get 1 DOT done per clinic week is too high and adds additional stress to the already busy clinic day when we’re running behind. I think that encouraging direct observation and feedback is important. I do not think that different forms or tools really make much of a difference. The time and attention of the preceptor is really what is important. Overall helpful in making observation take place. Having four different forms is almost irrelevant, but pulling the preceptor in before lengthy discussions with the patient does provide a valuable opportunity for feedback that occurs quite rarely in clinic
Scope	5	it is difficult to get these completed as it is hard to predict prior to the visit when the patients will have the appropriate issue for discussion, and it often feels awkward/is difficult to not have the discussion during the initial part of the visit (prior to precepting). While I appreciate the idea of being observed by preceptors and getting feedback on patient interactions, the DOTs are far too structured and have very limited applicability.
Sample faculty quotations *before* introduction of new tools		
Value	7	In doing this survey I am reminded of the saying ‘tis a poor carpenter that blames his tools.....our observations are guided by the form but are not limited to what is on the form. I think the evaluation form could do better in listing more specific behaviors we should be looking for. We can always free text our observations, but nice to have specific things that all the attendings look for. I find the mini-CEX is just a tool that promotes discussion rather than the tool itself giving feedback. I think this is fine as it creates a situation that promotes feedback
Artificial	4	I understand a measurement tool has to be used however I still think the interactions/observations are artificial; even residents who I know struggle are able to put on a reasonable performance while I am observing them. I think a combination of sitting in the room and letting the residents know I am there to enhance their experience with the patient and not to score … 5 mini cex [tools] would seem more valuable.
Sample faculty quotations *after* introduction of new tools		
Narrow scope	8	due to the specific nature of the tools it has been consistently difficult for my residents to get me in the room for the conversation without stopping their visit and coming to get me which can be awkward
Value	6	*I do feel that the new … forms are easier to use than the old ones - and I very much like the way they target specific high-yield communications topics that frequently arise in primary care clinic. Finding time to do the direct-observations continues to be an issue.*

^a^N = number responses

## Discussion

To our knowledge this is the first study to assess resident and preceptor perceptions of ACGME milestones-based direct observation tools. While our study shows that residents on average did not perceive increased benefits of milestones-based DOTs compared to the more general Mini-CEX direct observation and feedback tools, many of the qualitative comments demonstrate that residents appreciate direct observation in clinical teaching which is in line with other studies on resident attitude and direct observation [5–7]. (However, a number of residents also expressed dissatisfaction and inconvenience of using such tools in the continuity clinic setting. Given the time pressures inherent in the resident clinic these comments are understandable and similar barriers have been found in other studies [8]. Additionally, it is encouraging that some residents perceive substantial benefit derived from direct observation of these clinical encounters.

By contrast faculty preceptors reported significantly increased ratings using the new DOTs across a number of questions including increased satisfaction and ability to provide high quality feedback. Faculty comments reflected both the reality of time pressure and competing demands in the resident clinic setting as well as the unique benefits potentially derived from such direct observation. Other studies have also shown faculty satisfaction with direct observation tools in general though not specifically with milestones based tools [7, 9].

Strengths of the study include that responses were linked to specific respondents allowing us to compare respondents across surveys. Another strength was the use of a control group working in a separate area of clinical practice of the building, though with a similar work environment and staffing, which allowed us to account for temporal and other systemic factors that could have been influenced user perception using the tools. However, it is possible that there was contamination between both resident and preceptor groups since all were part of the same residency program and may have communicated about the tools they were using.

Limitations of the study include that it occurred at a single institution, thereby limiting our ability to understand how these tools would be received at other institutions with other sizes and types of trainees. Another issue to consider is the environment in which the new tools were implemented. Before initiation of the milestones-based direct observation tool program, general Mini-CEX tools were required by the residency program to be completed by residents and faculty twice per year and often were done in last minute fashion soon before the resident evaluation meeting with the program director. With the implementation of the new tools came an increased requirement in number of observations completed from twice per year to once every month. In addition, both residents and faculty received regular communication to complete the direct observations such as the monthly email reminders. As such it is possible that residents’ reactions to the new tools represented a response to the new system in place and the increased requirement to complete such tools rather than simply a response to the new tool itself. In most cases it is difficult to tease apart responses related to the milestones-based tools themselves from the act of direct observation and feedback. Additional limitations include that the tools in the study consist of only 4 clinical scenarios. While these are relatively common primary care clinical scenarios, the tools may be most applicable to clinical situations that match the topics of the tools. This may therefore limit the opportunity for direct observation to these certain scenarios. Next, this study was based solely on participant perceptions, and not objective measurements of clinical performance. Finally, the validity and reliability of the tools have not been determined.

The act of completing direct observation itself may matter as much or more than the tool used to give residents constructive formative feedback. Only by direct observation can faculty accurately assess certain clinical skills such as communication with patients or physical exam skills. Furthermore, some have pointed out that many physicians will not substantially improve such skills after residency training, thus emphasizing the importance of direct observation and feedback during training [10]. Getting faculty to do more direct observation is often cited as a challenge, thus making the tools easy to use and supporting faculty time to do so are both critical. [11]

As highlighted by Hauer et al., there are multiple steps to achieving successful implementation of a new tool for direct observation, including faculty development, assessing the reliability and validity of the tool, and the development of systems that facilitate observation [12]. Future directions for such work could include evaluating frequency of direct observation and strategies to lower barriers to use. This study evaluated the feasibility and acceptance of new direct observation tools and we were unable to completely evaluate and implement these other areas. However, we feel the face validity of the instruments is high as reflected by our qualitative findings that included few comments on content issues. In summary, many seemed to recognize the potential value of direct observation as well as its limitations. While residents perceived the new tools similarly to the mini-CEX tools, preceptors reported higher satisfaction and usefulness with the milestones-based DOTs. These new office based DOTs are a potentially useful addition to the tool box available for the assessment of clinical skills of medical trainees. Future research could examine what benefits trainees and faculty perceive by using DOTs as a clinical teaching method relative to more traditional summative end of rotation feedback, for example. As direct observation is recognized as increasingly important in making milestones-based assessments, accommodations should be made to facilitate its use and work to build perceptions of their appeal and importance among residents, such as identifying resident and faculty champions. This study sheds light on how to move forward in improving the resident and faculty experience with this important clinical teaching task.

## Conclusion

Both residents and faculty recognized the value and limitations of direct observation, and while residents perceived similarly the new DOTs to the more general mini-CEX tools, faculty reported comparatively increased satisfaction with the DOTs. The DOTs are a useful addition to the tool box available for the assessment of clinical skills of medical trainees, especially from the viewpoint of faculty preceptors.

## Additional file

Additional file 1: Samples of 4 DOT forms. (DOCX 27 kb)
