# Supplementary material for: Milestones-based direct observation tools in internal medicine resident continuity clinic
Source: BMC Med Educ. 2017 Dec 4;17:240. doi: 10.1186/s12909-017-1077-y (PMC5715641; doi:10.1186/s12909-017-1077-y)
Supplement: Additional file 1: — Samples of 4 DOT forms. (DOCX 27 kb) [file 12909_2017_1077_MOESM1_ESM.docx]

**Appendix**

**Milestones Checklist for Resident Lifestyle Change Counseling**

[smoking cessation, ETOH, dietary modification, exercise]

*Document whether you observe the resident possessing the specific skills.*

*Please be accurate in your assessment of the resident- It is* ***NOT*** *expected that a resident has mastered all these skills but rather that they receive feedback on their observed encounter. Proficiency in these skills is expected over 12-36 months of residency.*

|  | | Milestone | Yes | Partially | No | N/A |
| --- | --- | --- | --- | --- | --- | --- |
| PC-F3 | | Provide appropriate preventive care and teach patient regarding self-care |  |  |  |  |
| PC-F10 | | Customize care in the context of the patient’s preferences and overall health; negotiate the plan with the patient |  |  |  |  |
| MK-A5 | | Demonstrate sufficient knowledge to provide preventive care |  |  |  |  |
| ICS-A5 | | Utilize patient centered educational strategies |  |  |  |  |
| ICS-A4,6 | | Assessed the patient’s readiness to change |  |  |  |  |
| ICS-A4,6 | | Assessed the patient’s confidence in making the change |  |  |  |  |
| ICS-A2-3 | | Used language that the patient could understand |  |  |  |  |
| ICS-A2-3 | | Used appropriate non-verbal communication |  |  |  |  |
| ICS-A5 | | Assessed patient’s understanding at the end of the encounter |  |  |  |  |
| ICS-B2 | | Demonstrate sensitivity to differences in patients including race, culture, gender, sexual orientation, SES status, literacy, religion |  |  |  |  |
| SBP3 | | Recognizes that external factors influence a patient’s utilization of health care and may act as barriers to effective care |  |  |  |  |
|  | What did the resident do well? | | | | | |
|  | What is an area for improvement? | | | | | |

**Milestones Checklist for Resident Clinic Counseling Regarding an Age Appropriate Screening Test**

*Document whether you observe the resident possessing the specific skills.*

*Please be accurate in your assessment of the resident- It is* ***NOT*** *expected that a resident has mastered all these skills but rather that they receive feedback on their observed encounter. Proficiency in these skills is expected over 12-36 months of residency.*

***This does not assume the resident conducts the entire discussion. Please evaluate the portion the resident conducted; and if the preceptor intervened, please provide specific feedback why.***

|  | | Milestone | Yes | Partially | No | N/A |
| --- | --- | --- | --- | --- | --- | --- |
| MK-A5 | | Identified whether the patient met the guidelines |  |  |  |  |
| PC-F10 | | Conveyed to the patient the risks, benefits and alternative of the test |  |  |  |  |
| ICS-A2-4 | | Used language the patient could understand |  |  |  |  |
| ICS-A2 | | Used appropriate non-verbal communication to put the patient at ease and develop rapport with the patient |  |  |  |  |
| ICS-A4 | | Allowed time for questions |  |  |  |  |
| ICS-A4 | | Encouraged the patient to “repeat back” their understanding in their own words |  |  |  |  |
| ICS-A4 | | Engaged in collaborative, patient-centered decision making |  |  |  |  |
| SBP-D2 | | Considered costs of screening to the patient, society, and the health care system |  |  |  |  |
| P-I2 | | Recognize and manage conflict when patient values differ from own |  |  |  |  |
|  | | Preceptor needed to take over a portion of the discussion |  |  |  |  |
|  | If the preceptor took over a portion of the discussion, why did they need to? | | | | | |
|  | What did the resident do well? | | | | | |
|  | What is an area for improvement? | | | | | |

**Milestones Checklist for Explanation of Test Results to Patient**

*Document whether you observe the resident possessing the specific skills.*

*Please be accurate in your assessment of the resident- It is* ***NOT*** *expected that a resident has mastered all these skills but rather that they receive feedback on their observed encounter. Proficiency in these skills is expected over 12-36 months of residency.*

***This does not assume that the resident conducts the entire discussion. Please evaluate the portion the resident conducted; and if the preceptor intervened, please provide specific feedback why.***

|  | | Milestone | Yes | Partially | No | N/A |
| --- | --- | --- | --- | --- | --- | --- |
| MK-B1 | | Understood the indications for and basic interpretation of the test |  |  |  |  |
| ICS-A1 | | Explained the test result to the patient in a timely manner |  |  |  |  |
| ICS-A2 | | Used language the patient could understand |  |  |  |  |
| ICS-A2 | | Used appropriate nonverbal communication to put the patient at ease and develop rapport with patient |  |  |  |  |
| ICS-A5 | | Gave the patient the opportunity to ask questions |  |  |  |  |
| ICS-A5 | | Confirmed patient understanding of the results; asked the patient to repeat back in their own words. |  |  |  |  |
| PC-E2 | | Explained what happens next as a result of the test result |  |  |  |  |
| ICS-A4 | | Engaged patient/family in shared decision making |  |  |  |  |
| P-B1 | | Demonstrated empathy and compassion to the patient |  |  |  |  |
| PC-F5 | | Was able to independently manage the encounter (explain the results and answer all questions to the patient and attending’s satisfaction) |  |  |  |  |
|  | If the preceptor needed to take over a portion of the discussion, why did they need to do so? | | | | | |
|  | What did the resident do well? | | | | | |
|  | What is an area for improvement? | | | | | |

**Milestones Checklist for Resident Clinic Controlled Substance Contract Discussion**

*Document whether you observe the resident possessing the specific skills.*

*Please be accurate in your assessment of the resident- It is* ***NOT*** *expected that a resident has mastered all these skills but rather that they receive feedback on their observed encounter. Proficiency in these skills is expected over 12-36 months of residency.*

***This does not assume the resident conducts the entire discussion. Please evaluate the portion the resident conducted; and if the preceptor intervened, please provide specific feedback why****.*

|  | | Milestone | Yes | Partially | No | N/A |
| --- | --- | --- | --- | --- | --- | --- |
| PBLI-E3 | | Communicated the risks and benefits of the controlled substance contract |  |  |  |  |
| P-B1 | | Demonstrated empathy and compassion to the patient |  |  |  |  |
| P-B2 | | Demonstrated a commitment to relieve pain and suffering |  |  |  |  |
| ISC-A2 | | Used language the patient could understand |  |  |  |  |
| ISC-A2 | | Effectively used nonverbal communication to put the patient at ease and develop rapport with the patient |  |  |  |  |
| ICS-A3 | | Built a therapeutic relationship |  |  |  |  |
| MK-A3 | | Demonstrates sufficient knowledge and conduct the discussion to the satisfaction of both the patient and the preceptor |  |  |  |  |
|  | | Preceptor needed to take over a portion of the discussion |  |  |  |  |
|  | If the preceptor needed to take over a portion of the discussion, why did they need to do so? | | | | | |
|  | What did the resident do well? | | | | | |
|  | What is an area for improvement? | | | | | |

**Explanation of tools:**

Each tool is based on the observation of a specific clinical scenario (EPA=entrustable professional activity) and the curricular milestones that may be addressed when engaging in the EPA. The resident is judged specifically on each of the curricular milestones listed in the tool- the left column references the milestone (e.g. MK-B1= Medical knowledge, curricular milestone B1) . Thus the specific assessments are made against milestones and the preceptor is given the choice of yes, no, partially or not applicable when answering whether each milestone is met. There is space at the bottom of each tool to elaborate if needed. The competency is not addressed differently at different stages or year of training.
